# Prognostic analysis of hepatocellular carcinoma with macrovascular invasion after liver resection and a successful case of conversion therapy

**DOI:** 10.3389/fsurg.2022.1042431

**Published:** 2022-11-07

**Authors:** Mengling Ji, Hao Zou, Baojun Shu, Guoping Liu, Bingyuan Zhang, Zejiang Xu, Fanfan Pang, Mengxing Cheng, Yafei Sun, Ting Du, Chuandong Sun, Chengzhan Zhu

**Affiliations:** ^1^ Department of Hepatobiliary and Pancreatic Surgery, The Affiliated Hospital of Qingdao University, Qingdao, China; ^2^ Department of Operation Room, The Affiliated Hospital of Qingdao University, Qingdao, China; ^3^ Department of Interventional Radiology, The Affiliated Hospital of Qingdao University, Qingdao, China; ^4^ Medical Department, Yidu Cloud (Beijing) Technology Co., Ltd., Beijing, China; ^5^ Shandong Key Laboratory of Digital Medicine and Computer Assisted Surgery, The Affiliated Hospital of Qingdao University, Qingdao, China

**Keywords:** hepatocellular carcinoma, macrovascular invasion, liver resection, prognosis, conversion therapy

## Abstract

**Objective:**

Macrovascular invasion (MVI) is an important factor leading to poor prognosis in hepatocellular carcinoma (HCC). Liver resection may offer favorable prognosis for selected patients with HCC. This study aimed to analyze the prognostic factors of HCC with MVI after liver resection as well as demonstrate a case of conversion therapy in an HCC patient with portal vein tumor thrombus (PVTT).

**Methods:**

A total of 168 HCC patients with MVI who underwent primary liver resection at the Affiliated Hospital of Qingdao University between January 2013 and October 2021 were enrolled in the study. Clinicopathological data were collected retrospectively. Univariate and multivariate regression analyses were used to investigate the risk factors influencing recurrence and overall survival. Additionally, conversion therapy with drug-eluting bead transarterial chemoembolization (D-TACE), and sorafenib plus sintilimab treatment was performed in an HCC patient with PVTT.

**Results:**

Among the 168 patients with HCC, 11 were diagnosed with hepatic vein tumor thrombosis, and the rest were diagnosed with PVTT. The 1-year disease-free survival rate was 37.5%, and the 3-year overall survival rate was 52.7%. Univariate and multivariate regression analyses revealed that HBsAg positivity, alpha-fetoprotein (AFP) level ≥400 ng/ml, liver capsule invasion, and tumor number ≥2 were independent prognostic factors for tumor recurrence, whereas HBsAg positivity was an independent risk factor for overall survival. Postoperative prophylactic medication did not significantly prolong the recurrence time. The median survival time (MST) after tumor recurrence was 13.4 months. In the patient treated with conversion therapy, the tumor gradually shrank and was eventually surgically resected.

**Conclusions:**

This study identified the independent prognostic and risk factors associated with recurrence and overall survival in HCC patients with MVI. Additionally, we successfully performed conversion therapy in an HCC patient with PVTT. The findings would help identify patients at high risk of recurrence and indicate that combined therapy may prolong the survival of HCC patients with PVTT.

## Introduction

Hepatocellular carcinoma (HCC) is the fifth most prevalent malignancy in China (1). More than 50% of patients are diagnosed with advanced HCC, of which macrovascular invasion (MVI) is one of the most common signs (2). The incidence of portal vein tumor thrombus (PVTT), which is the main type of MVI, in patients with HCC is 44%–62.2% (3). PVTT formation promotes distant metastasis and induces portal hypertension, leading to a poor prognosis (4). Patients with untreated HCC with PVTT have a 2.7–4.0-month median survival time (5, 6). Although targeted and immune therapies have greatly improved the prognosis of advanced HCC, the current approach for treating HCC patients with PVTT remains controversial.

Most guidelines recommend non-surgical treatments, including transarterial chemoembolization (TACE) or sorafenib, as the first-line treatment for HCC patients with PVTT (7). However, some patients with HCC and MVI can undergo surgical resection. A meta-analysis comparing the prognosis of surgical resection with non-surgical resection indicated that TACE or other non-surgical treatments were inferior to surgical resection in terms of overall survival (OS) (8). Thus, the latest guidelines in China recommend that surgical resection be considered if the tumor thrombus can be completely removed during the operation, followed by TACE, portal vein chemotherapy, or other systemic treatments to prevent recurrence (8, 9). Neoadjuvant three-dimensional radiotherapy can also be performed preoperatively (9, 10). With the progress in targeted therapy and immunotherapy, more options are available for the treatment of HCC (11). The combination of different therapies has been shown to be secure and more useful in patients with HCC with MVI (12). The strategy of combining different treatments should be arranged according to the risk of recurrence or prolonged survival; however, such strategies have not yet been established.

In this study, we retrospectively analyzed patients who underwent hepatectomy at our hospital and explored the independent risk factors for disease-free survival (DFS) and OS. We report a successful case of conversion therapy with a combination of D-TACE, sorafenib plus sintilimab (PD-1) treatment and surgical resection.

## Materials and methods

### Study population

This retrospective study included 168 HCC patients with MVI who underwent liver resection at the Affiliated Hospital of Qingdao University between January 2013 and October 2021. The criteria for selecting patients for hepatectomy were as follows: (1) type I or II PVTT, (2) resectable hepatic vein tumor thrombosis, (3) PVTT after liver resection with Child-Pugh class A or B liver function (score ≤7), and (4) absence of extrahepatic metastases or other associated malignancies. We collected clinicopathological data and information on survival outcomes of all patients. The exclusion criteria were as follows: (1) incomplete follow-up information and (2) incomplete tumor resection.

Ethics approval for this study was provided by the ethics committee of the Affiliated Hospital of Qingdao University.

### Classification of PVTT

The diagnostic criteria for PVTT were based on typical preoperative imaging studies and confirmed by postoperative histopathological examination (13). According to Cheng's classification (14), there are four different types of PVTT: type I, which invades the segmental branches of the portal vein or higher; type II, which invades the right or left portal vein; type III, which invades the main portal vein; and type IV, which invades the main portal and superior mesenteric veins.

### Patient follow-up

All patients who received treatment for HCC at our hospital underwent outpatient follow-up after liver resection once a month for the first three months and then every two–three months until death or withdrawal from the follow-up. Serum alpha-fetoprotein (AFP) level analysis, liver function tests, whole blood counts, abdominal ultrasound, and enhanced CT were used for follow-up examinations.

### Statistical analysis

The primary endpoint, which was OS, was determined as the time from the procedure until death or the last follow-up; DFS was calculated as the time from the procedure to recurrence. The chi-square test or Fisher's exact test was used to compare categorical variables, which are represented as rates or constituent ratios. Univariate and multivariate logistic regression analyses were used to explore variables that showed an independent relationship with recurrence. Survival curves were constructed using the Kaplan-Meier method and compared using the log-rank test. Statistical significance was set at *P* < 0.05. Statistical analysis was performed using IBM SPSS Statistics version 20 (SPSS, Chicago, IL, United States).

## Results

### Patient characteristics

**Figure 1:**
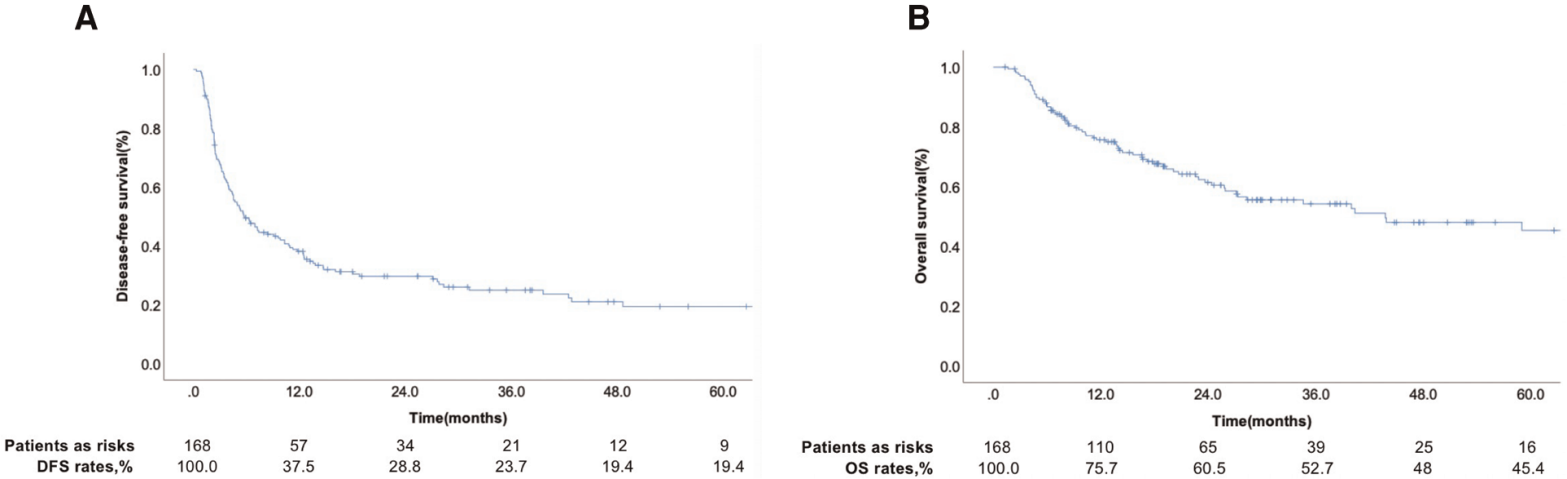
The disease-free survival (**A**) and overall survival (**B**) rate of patients with MVI..

Among the 168 patients, 11 were diagnosed with HCC with hepatic vein tumor thrombosis and the rest with HCC with PVTT. The curves for DFS and OS are shown in Figures 1A, B. Among the patients, 124 patients had recurrence and 44 patients did not show recurrence during the follow-up. The 1-year DFS rate of the entire cohort was 37.5%. We seperated the patients into two groups according to whether patients occured tumor recurrence during follow-up and comprared the risk factor related with tumor recurrence. Table 1 showed that the tumor number ≥ 2 was related with tumor recurrence.. The 1-year and 3-year accumulating OS rates were approximately 75.7% and 52.7%, respectively.

**Table 1:** Correlation of clinicopathological factors with tumor recurrence.

Variable	No recurrence (*n* = 44)	Recurrence (*n* = 124)	*P*
Age (years)	56.57 ± 8.963	55.78 ± 9.671	0.638
Sex			0.089
Female	10 (22.7%)	15 (12.1%)	
Male	41 (80.4%)	102 (87.2%)	
Alcohol abuse			0.579
Yes	16 (36.4%)	51 (41.1%)	
No	28 (63.6%)	73 (58.9%)	
Tumor number			0.006
Single	43 (97.7%)	100 (80.6%)	
≥2	1 (2.3%)	24 (19.4%)	
Tumor diameter			0.63
>5 cm	31 (70.5%)	92 (74.2%)	
≤5 cm	13 (29.5%)	32 (25.8%)	
Live cirrhosis			0.469
Yes	34 (77.3%)	102 (82.3%)	
No	10 (22.7%)	22 (17.7%)	
Tumor differentiation			0.385
I/II	17 (38.6%)	39 (31.5%)	
III/IV	27 (61.4%)	85 (68.5%)	
Liver capsule invasion			0.034
Yes	17 (38.6%)	71 (57.3%)	
No	27 (61.4%)	53 (42.7%)	
Tumor satellite			0.944
Yes	15 (34.1%)	43 (34.7%)	
No	29 (65.9%)	81 (65.3%)	
Microvascular invasion			0.77
M0	9 (20.5%)	28 (22.6%)	
M1/M2	35 (79.5%)	96 (77.4%)	
BDTT			0.589
Yes	4 (9.1%)	15 (12.1%)	
No	40 (90.9%)	109 (87.9%)	
Surgical margin			0.691
<1 cm	16 (36.4%)	41 (33.1%)	
≥1 cm	28 (63.6%)	83 (66.9%)	
Intraoperative blood loss			0.04
>200 ml	27 (73.0%)	91 (87.5%)	
≤200 ml	10 (27%)	13 (12.5%)	
Intraoperative blood transfusion			0.445
Yes	5 (11.4%)	20 (16.1%)	
No	39 (88.6%)	104 (83.9%)	
T-Bil (µmol/L)			0.713
>17.1	22 (50%)	66 (53.2%)	
≤17.1	22 (50%)	58 (46.8%)	
ALT (U/L)			0.036
>44	10 (22.7%)	50 (40.3%)	
≤44	34 (77.3%)	74 (59.7%)	
GGT (U/L)			0.079
>64	21 (47.7%)	78 (62.9%)	
≤64	23 (52.3%)	46 (37.1%)	
PT (s)			0.34
>13	7 (15.9%)	13 (10.5%)	
≤13	37 (84.1%)	111 (89.5%)	** **
Types of PVTT			0.592
I	18 (45%)	47 (40.2%)	** **
II	22 (55%)	70 (59.8%)	** **
AFP (ng/ml)	** **	** **	0.699
≤400	21 (47.7%)	55 (44.4%)	** **
>400	23 (52.3)	65 (55.6%)	
Perioperative TACE	** **	** **	0.252
Yes	19 (43.2%)	66 (53.2%)	** **
No	25 (56.8%)	58 (46.8%)	

BDTT, bile duct tumor thrombus; T-Bil, total bilirubin; ALT, alanine aminotransferase; GGT, *γ*-glutamyltransferase; PT, prothrombin; AFP, alpha-fetoprotein; TACE, transarterial chemoembolization.

### Clinicopathological factors related to DFS and OS

**Figure 2:**
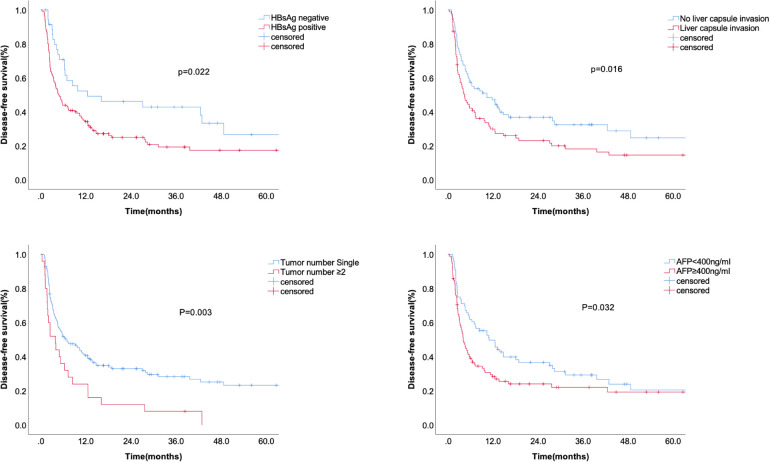
The disease-free survival according to HBsAg, liver capsule invasion, AFP level, and tumor number.

Kaplan-Meier analysis identified significant factors related to DFS (Figure 2). Univariate analysis showed that HBsAg positivity, AFP level ≥400 ng/ml, liver capsule invasion, and tumor number ≥2 were risk factors for tumor recurrence after hepatectomy. Multivariate analysis showed that HBsAg positivity (OR = 1.667, 95% CI: 1.055–2.634, *P* = 0.029), AFP level ≥400 ng/ml (OR = 1.606, 95% CI: 1.121–2.300, *P* = 0.01), liver capsule invasion (OR = 1.496, 95% CI: 1.045–2.143, *P* = 0.028), and tumor number ≥2 (OR = 2.101, 95% CI: 1.337–3.302, *P* = 0.001) were independent risk factors for tumor recurrence (Table 2). A non-significant difference was observed between type I and type II PVTT (*P* = 0.263).

**Table 2:** Univariate and multivariate analysis of prognostic factors that related with tumor recurrence.

Variable	Univariate (*P*)	Multivariate (*P*)	OR (95% CI)
Age (>60 vs ≤60)	0.865		
Sex (Male vs Female)	0.245		
Alcohol abuse (Y/N)	0.754		
HBsAg (Y/N)	**0**.**022**	**0**.**029**	**1**.**667** (**1**.**055–2**.**634)**
Tumor number (Single/≥2)	**0**.**003**	**0**.**001**	**2**.**101** (**1**.**337–3**.**302)**
Tumor diameter >5 cm (Y/N)	0.101		
Live cirrhosis (Y/N)	0.981		
Tumor differentiation (I/II vs III/IV)	0.241		
Liver capsule invasion (Y/N)	**0**.**016**	**0**.**028**	**1**.**496** (**1**.**045–2**.**143)**
Tumor satellite (Y/N)	0.472		
Microvascular invasion (M0 vs M1/M2)	0.645		
BDTT (Y/N)	0.46		
Surgical margin <1 cm (Y/N)	0.54		
Intraoperative blood loss > 200 ml (Y/N)	0.114		
Intraoperative blood transfusion (Y/N)	0.332		
TBIL >17.1 umol/L (Y/N)	0.732		
ALT >44 U/L (Y/N)	0.089		
GGT >64 U/L (Y/N)	0.064		
PT >13 s (Y/N)	0.644		
Types of PVTT (I vs II)	0.263		
AFP ≥ 400 ng/ml (Y/N)	**0**.**032**	**0**.**01**	**1**.**606** (**1**.**121–2**.**300)**
Perioperative TACE	0.389	** **	** **

HBsAg, type B hepatitis surface antigen; MVI, microvascular invasion; BDTT, bile duct tumor thrombus; T-Bil, total bilirubin; ALT, alanine aminotransferase; GGT, *γ*-glutamyltransferase; PT, prothrombin; AFP, alpha-fetoprotein; TACE, transarterial chemoembolization. OR, odds ratio; CI, confidence interval.

**Table 3:** Correlation of clinicopathological factors with overall survival.

Variable	Alive (*n* = 98)	Dead (*n* = 70)	*P*
Age (years)	56.48 ± 9.298	55.3 ± 9.732	0.428
Sex			0.052
Female	19 (9.4%)	6 (8.6%)	
Male	79 (80.6%)	64 (91.4%)	
Alcohol abuse			0.324
Yes	36 (36.7%)	31 (44.3%)	
No	62 (63.3%)	39 (55.7%)	
Tumor number			0.014
Single	89 (90.8%)	54 (77.1%)	
≥ 2	9 (9.2%)	16 (22.9%)	
Tumor diameter			0.536
> 5 cm	70 (71.4%)	53 (75.7%)	
≤ 5 cm	28 (28.6%)	17 (24.3%)	
Live cirrhosis			0.352
Yes	77 (78.6%)	59 (84.3%)	
No	21 (21.4%)	11 (15.7%)	
Tumor differentiation			0.825
I/II	32 (32.7%)	24 (34.3%)	
III/IV	66 (67.3%)	46 (65.7%)	
Liver capsule invasion			0.095
Yes	46 (46.9%)	42 (60%)	
No	52 (53.1%)	28 (40%)	
Tumor satellite			0.297
Yes	37 (37.1%)	21 (30%)	
No	61 (62.2%)	49 (70%)	
Microvascular invasion			0.176
M0	18 (18.4%)	19 (27.1%)	
M1/M2	80 (81.6%)	51 (72.9%)	
BDTT			0.344
Yes	13 (13.3%)	6 (8.6%)	
No	85 (86.7%)	64 (91.4%)	
Surgical margin			0.934
<1 cm	33 (33.7%)	24 (34.3%)	
≥1 cm	65 (66.3%)	46 (65.7%)	
Intraoperative blood loss			0.069
>200 ml	63 (78.7%)	55 (90.2%)	
≤200 ml	17 (21.3%)	6 (9.8%)	
Intraoperative blood transfusion			0.798
Yes	14 (14.3%)	11 (15.7%)	
No	84 (85.7%)	59 (84.3%)	
T-Bil (µmol/L)			0.175
>17.1	47 (48%)	41 (58.6%)	
≤17.1	51 (52%)	29 (41.4%)	
ALT (U/L)			0.102
>44	30 (30.6%)	30 (42.9%)	
≤44	68 (69.4%)	40 (57.1%)	
GGT (U/L)			0.233
>64	54 (55.1%)	45 (64.3%)	
≤ 64	44 (44.9%)	25 (35.7%)	
PT (s)			0.107
>13	15 (15.3%)	5 (7.1%)	
≤13	83 (84.7%)	65 (92.9%)	
Types of PVTT			0.106
I	43 (46.7%)	22 (33.8%)	
II	49 (53.3%)	43 (66.2%)	
AFP (ng/ml)			0.173
≤400	40 (40.8%)	55 (51.4%)	
>400	58 (59.2%)	65 (48.6%)	
Perioperative TACE			
Yes	52 (53.1%)	33 (47.1%)	0.449
No	46 (46.9%)	37 (52.9%)	

BDTT, bile duct tumor thrombus; T-Bil, total bilirubin; ALT, alanine aminotransferase; GGT, *γ*-glutamyltransferase; PT, prothrombin; AFP, alpha-fetoprotein; TACE, transarterial chemoembolization.

**Figure 3:**
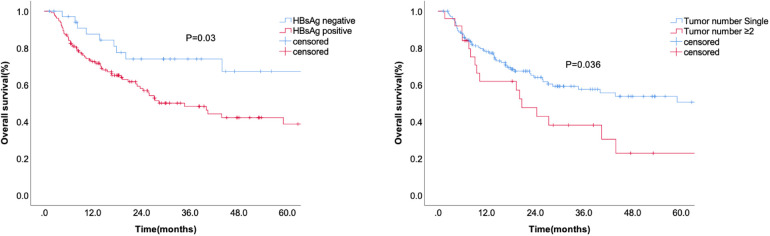
The overall survival according to HBsAg and tumor number.

Table 3 showed the correlation analysis of clinicopathological factors with overall survival. Furthermore, Kaplan-Meier analysis identified the significant factors associated with OS (Figure 3). Univariate analysis showed that HBsAg positivity and tumor number ≥2 were significantly correlated with OS (Table 4). Multivariate analysis showed that HBsAg positivity (OR = 2.007, 95% CI: 1.024–3.931, *P* = 0.042) was an independent risk factor for OS.

**Table 4:** Univariate and multivariate analysis of prognostic factors that related with survival.

Variable	Univariate (*p*)	Multivariate (*p*)	OR (95% CI)
Age (>60 vs ≤60)	0.896		
Sex (Male vs Female)	0.192		
Alcohol abuse (Y/N)	0.94		
HBsAg (Y/N)	**0** **.** **03**	**0** **.** **042**	2.007 (1.024–3.931)
Tumor number (Single/≥2)	**0**.**036**	0.054	1.736 (0.991–3.042)
Tumor diameter > 5 cm (Y/N)	0.256		
Live cirrhosis (Y/N)	0.952		
Tumor differentiation (I/II vs III/IV)	0.902		
Liver capsule invasion (Y/N)	0.066		
Tumor satellite (Y/N)	0.502		
Mcrovascular invasion (M0 vs M1/M2)	0.816		
BDTT (Y/N)	0.644		
Surgical margin <1 cm (Y/N)	0.667		
Intraoperative blood loss >200 ml (Y/N)	0.157		
Intraoperative blood transfusion (Y/N)	0.849		
TBIL > 17.1 umol/L (Y/N)	0.17		
ALT > 44 U/L (Y/N)	0.115		
GGT > 64 U/L (Y/N)	0.166		
PT > 13 s (Y/N)	0.578		
Types of PVTT (I vs II)	0.123		
AFP ≥ 400 ng/ml (Y/N)	0.92	** **	
Perioperative TACE	0.342		

HBsAg, type B hepatitis surface antigen; BDTT, bile duct tumor thrombus; T-Bil, total bilirubin; ALT, alanine aminotransferase; GGT, *γ*-glutamyltransferase; PT, prothrombin; AFP, alpha-fetoprotein; TACE, transarterial chemoembolization. OR, odds ratio; CI, confidence interval.

### Effect of targeted therapy on patient prognosis

**Figure 4:**
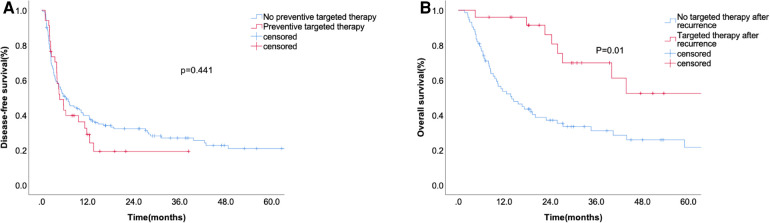
The disease-free survival according to preventive targeted therapy before liver resection (**A**) and overall survival (**B**) according to whether accepting targeted therapy after recurrence.

We analyzed the effects of targeted therapy on DFS and OS. Postoperative prophylactic medication did not significantly prolong the recurrence time (Figure 4A). The median surveillance time of OS after recurrence was 33 months in the targeted therapy group and 7.3 months in the non-targeted therapy group (Figure 4B).

### Successful case of conversion therapy

**Figure 5:**
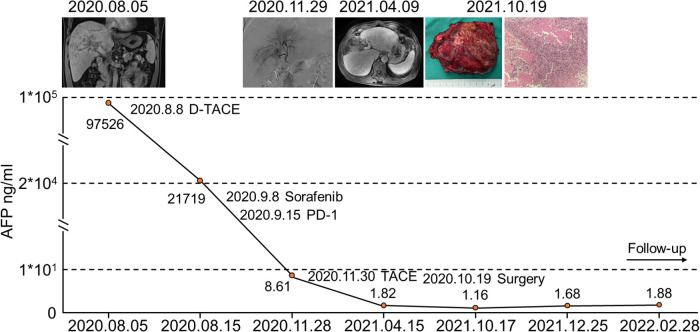
A successful case of conversion therapy.

We report a successful case of conversion therapy with drug-eluting bead-TACE (D-TACE), sorafenib plus sintilimab (PD-1) treatment and liver resection. A 49-year-old male was admitted to our institution with a complaint of an intrahepatic mass. Enhanced CT of the upper abdomen indicated HCC with type II PVTT. The pretreatment AFP level was 97,526 ng/ml. First, D-TACE was performed, followed by sorafenib plus sintilimab (PD-1) targeted immunotherapy. The tumor gradually shrank, and the patient eventually underwent surgical resection. The entire treatment process and the changes in AFP levels are shown in Figure 5. After combined conversion therapy, the patient successfully underwent surgery.

## Discussion

One of the most important adverse factors affecting the prognosis of patients with HCC is macrovascular invasion (MVI), particularly PVTT. Although several new treatment methods have been developed for HCC patients with MVI, selecting the best treatment remains controversial. With the progress in surgical technology, surgical resection of HCC with MVI has been identified as safe and effective (15). However, owing to the high recurrence rate of HCC with MVI, the postoperative long-term survival prognosis of these patients is poor (16). This study analyzed the risk factors that affect prognosis after hepatectomy, providing clues for improving patient prognosis.

Surgical resection is an effective treatment for patients with HCC and MVI. A propensity-score matched study in a large North American multi-institutional cohort showed that any surgical management was associated with improved survival (17). In another Japanese cohort including 100 HCC patients with Vp3 or Vp4 PVTT, hepatectomy was an effective treatment, with a median survival time of 14.5 months (18). A nationwide study in China, including 19 hospitals, found that the actual 3-year survival rate for patients with HCC with PVTT after hepatectomy was 11.7% (19). One study investigated the optimal treatment for patients with different types of PVTT, indicating that surgical resection was the best option for type I or II PVTT, TACE was recommended for type III PVTT, and sorafenib was more appropriate for type IV PVTT (20). Moreover, liver transplantation seems to offer a better prognosis for HCC with type I PVTT than liver resection, especially in patients with AFP levels >200 ng/ml (21). Patients underwent living donor liver transplantation after downstaging PVTT using stereotactic body radiotherapy (SBRT) and tumor ablation (with transarterial chemo- or radio-embolization). After successful downstaging, HCC patients with PVTT can have prolonged survival with living donor liver transplantation (22).

As for the prognostic risk factors, various clinicopathological risk factors, including AFP level >400 ng/ml, extent of PVTT, and tumor diameter >5 cm, and almost all risk factors related to HCC patient prognosis, were related to actual long-term survival in a Chinese nationwide study (19). In a single center in China, AFP level, ascites, and total bilirubin level were independent risk factors for poor short-term survival, whereas tumor differentiation and AFP level were associated with long-term survival (23). Another study found that tumor recurrence occurred after liver resection in 82.1% of patients with HCC with PVTT, and the median time for tumor recurrence was 4.1 months. PVTT in the main portal trunk and maximum tumor size ≥5 cm were the major prognostic factors influencing HCC recurrence following liver resection (24). In our study, the median recurrence was 13.4 months. and HBsAg positivity, AFP level ≥400 ng/ml, invasion of the liver capsule, and tumor number ≥2 were independent prognostic factors for recurrence, whereas HBsAg positivity was an independent risk factor of overall survival.

Furthermore, we have reported a successful case of conversion therapy with combined D-TACE, sorafenib plus sintilimab (PD-1) treatment and surgical resection. TACE is an effective and safe treatment for patients with advanced HCC. A meta-analysis examined the role of TACE in the treatment of HCC with PVTT, and the median OS was 8 (range: 5–15) months (25). The combination of TACE and targeted therapy can improve the prognosis of patients with advanced HCC. In patients with advanced HCC and PVTT, TACE plus lenvatinib was safe, well-tolerated, and more effective than TACE plus sorafenib (7). Patients with unresectable HCC may benefit from hepatic arterial infusion of oxaliplatin plus raltitrexed, regardless of whether they have PVTT (26). In patients with HCC and PVTT, cTACE-HAIC was superior to cTACE alone in terms of OS and PFS (27). A randomized phase 3 trial showed a higher response rate and longer median progression-free survival with sorafenib plus HAIC than sorafenib alone (28). Moreover, radiotherapy has been confirmed as an effective method for regressing tumor thrombi in the portal vein (10). Complete regression was achieved following radiotherapy for Vp3/Vp4 HCC in 3.6% of cases, partial regression in 50.2% of cases, and stable illness in 25.6% of cases, with an MST of 10.6 months, demonstrating the effectiveness of radiotherapy (29). A randomized multicenter controlled study showed that neoadjuvant radiotherapy provided considerably better postoperative survival outcomes than surgery alone, wherein 17 patients (20.7%) in the neoadjuvant radiation group experienced partial remission (10). The combination of SBRT and tumor ablation successfully downstaged 63% (27/43) of patients (22). Following partial hepatectomy ± thrombectomy, patients with HCC and PVTT who received postoperative IMRT experienced considerably better overall survival outcomes than those without IMRT (30). A prospective, controlled, multicenter study showed that TACE-125 iodine treatment greatly increased the survival of patients with type II PVTT and HCC, particularly subtype IIa, with few adverse events (31). Imbrave 150 has demonstrated similar effectiveness to T + A (32). In our study, liver resection was successfully performed after treatment with D-TACE and sorafenib plus PD-1.

Our study had several limitations. First, this was a retrospective study conducted at a single center and we included patients with long-time follow up. Second, as a single-center study, targeted therapy may be affected by the bias of subjects and the experience and preferences of surgeons. Multicenter and prospective studies should be designed to further explore the strategies for treating HCC with MVI.

In conclusion, we analyzed the prognostic factors for patients with HCC and MVI and demonstrated that the use of targeted therapy after recurrence can improve the overall survival of patients. We anticipate that the findings of this study will provide clues for identifying patients at high risk of recurrence and provide evidence of successful targeted treatment in patients with recurrence.

## Data Availability

The original contributions presented in the study are included in the article/Supplementary Material, further inquiries can be directed to the corresponding author/s.
